# Comparative Analysis of Genomics and Proteomics in *Bacillus thuringiensis* 4.0718

**DOI:** 10.1371/journal.pone.0119065

**Published:** 2015-03-17

**Authors:** Jie Rang, Hao He, Ting Wang, Xuezhi Ding, Mingxing Zuo, Meifang Quan, Yunjun Sun, Ziquan Yu, Shengbiao Hu, Liqiu Xia

**Affiliations:** College of Life Science, Hunan Normal University, Hunan Provincial Key Laboratory of Microbial Molecular Biology-State Key Laboratory Breeding Base of Microbial Molecular Biology, Changsha, China; Instituto de Biotecnología, Universidad Nacional Autónoma de México, MEXICO

## Abstract

*Bacillus thuringiensis* is a widely used biopesticide that produced various insecticidal active substances during its life cycle. Separation and purification of numerous insecticide active substances have been difficult because of the relatively short half-life of such substances. On the other hand, substances can be synthetized at different times during development, so samples at different stages have to be studied, further complicating the analysis. A dual genomic and proteomic approach would enhance our ability to identify such substances, and particularily using mass spectrometry-based proteomic methods. The comparative analysis for genomic and proteomic data have showed that not all of the products deduced from the annotated genome could be identified among the proteomic data. For instance, genome annotation results showed that 39 coding sequences in the whole genome were related to insect pathogenicity, including five *cry* genes. However, Cry2Ab, Cry1Ia, Cytotoxin K, Bacteriocin, Exoenzyme C3 and Alveolysin could not be detected in the proteomic data obtained. The sporulation-related proteins were also compared analysis, results showed that the great majority sporulation-related proteins can be detected by mass spectrometry. This analysis revealed Spo0A~P, SigF, SigE(+), SigK(+) and SigG(+), all known to play an important role in the process of spore formation regulatory network, also were displayed in the proteomic data. Through the comparison of the two data sets, it was possible to infer that some genes were silenced or were expressed at very low levels. For instance, found that *cry2Ab* seems to lack a functional promoter while *cry1Ia* may not be expressed due to the presence of transposons. With this comparative study a relatively complete database can be constructed and used to transform hereditary material, thereby prompting the high expression of toxic proteins. A theoretical basis is provided for constructing highly virulent engineered bacteria and for promoting the application of proteogenomics in the life sciences.

## Introduction

*Bacillus thuringiensis* (*B*. *thuringiensis*) is a widely used biopesticide that produced various insecticidal active substances during its life cycle. As of this writing, a dozen different functional bioactive components have been found in *B*. *thuringiensis* [1–5]. When some highly virulent *B*. *thuringiensis* strain are isolated and identified by conventional methods, such as, aflinity chromatography or isoelectric focusing electrophoresis, researchers often fail to obtain a comprehensive understanding of the insecticidal active substances. Separation and purification of numerous insecticide active substances have been difficult because of the relatively short half-life of such substances. What is more important that conventional methods are only among the many research tools, and such techniques fail to sufficiently, comprehensively, and accurately analyze *B*. *thuringiensis* active substances with insecticidal activity. And studies showed recently that application of genomics and proteomics respectively would enhance our understanding for these substances. In 2001, the first human genome sketch was successfully published by researchers, and this study prompted further research on these insecticidal active substances at the genome level [6, 7]. Strain CT-43 was used for the first complete whole genome sequencing of insecticidal crystal (Cry) protein gene, and a full-length genome of 6.15 Mb was obtained. This strain produced at least six kinds of toxin proteins [8]. And now, 15 *B*. *thuringensis* have completed the sequencing of whole genome, 15 *B*. *thuringensis* have completed the assembling of chromosome. In addition, 11 *B*. *thuringensis* are ongoing splicing (http://www.ncbi.nlm.nih.gov/genome/genomes/486?).

Some *B*. *thuringiensis* genomes that range from 5.31 Mb to 6.77 Mb in size (including one to multiple circle plasmids) have become known with the publication of most *B*. *thuringiensis* genome sequences, the guanine-cytosine content (GC) content of such genomes is between 34.5% and 35.6% [9–11]. The sequenced *B*. *thuringiensis* can enable rapid detection of genes that encode proteins with potential insecticidal activity at the genome level. The published genome sequence of *B*. *thuringiensis serovar israelensis* strain HD-789 contains seven Cry toxin genes, three Cyt toxin genes, and one Hemagglutinin gene [12]. The genome sequence of the *B*. *thuringiensis subsp*. *thuringiensis* strain IS5056 contains nine Cry toxin genes [13].

Research on *B*. *thuringiensis* genome accelerates the detection of important pathogenic genes and related regulatory factors. However, information on the proteins that were obtained from the genome is limited. Protein modification, protein–protein interactions, and protein interaction with other molecular components are not directly determined from the genome sequence. Therefore, to obtain a deeper understanding of functions and expression of pathogenic genes, the proteins should be studied at the level of overall dynamics and networks, collectively known as proteomic research. Wu D et al. studied *B*. *thuringiensis* strain YBT-1520 proteomics under high temperature conditions, and the expressed proteins comprised sporulation and insecticidal Cry proteins, the expressions of such proteins were reduced after treatment at 42°C [14]. Huang et al. reported the expression of other proteins in *B*. *thuringiensis* by studying proteomics at different growth phases using liquid chromatography–mass spectrometry/mass spectrometry (LC–MS/MS) and carried out preliminary analysis for the associated proteins [15]. Traditionally, LC is the most widely used proteomic method for analyzing complex protein mixtures prior to MS analysis [16, 17]. This method exhibits strong ability to identify, modify, quantify, and localize proteins [18, 19]. Therefore, mass spectrometry-based proteomic methods could be widely applied in insecticidal activity matters research.

Based on the above mentioned data, the methods of genomics and proteomics can be applied in studying *B*. *thuringiensis* proteins with insecticidal activity and with different characteristics. However, information is lacking on the combination of genomics and proteomics in the study of *B*. *thuringiensis* proteins with insecticidal activity. Mohamed A.I et al. conducted genomic and proteomic studies on such proteins and have developed a new research methodology in proteogenomics that can be used in various studies [20–23]. Therefore, this methodology can be used not only in discovering insecticidal protein genes and regulatory factors, but also in analyzing the expression of pathogenicity genes.

Our laboratory has contributed to the research on *B*. *thuringiensis* pesticides. In the present study, we performed whole genome sequencing to analyze high virulence *B*. *thuringiensis* strain 4.0718 and to search for pathogenicity genes related to pesticidal activity. Mass spectrometry analysis of *B*. *thuringiensis* proteome was performed for different periods to obtain differential expression of proteins. The first thing we searched the virulence gene from the *B*. *thuringiensis* genome data. And then compared the MS-based proteomic data with genome annotation data, analyzed spore formation and regulation, predicted insecticidal Cry protein interaction network, and verified the genome annotation results. The research results from the whole genome sequencing and MS analysis of the proteome could provide a theoretical basis for constructing highly virulent, genetically engineered bacteria and for applying proteogenomics in the life sciences.

## Materials and Methods

### Bacterial strains and growth conditions

The organism used in this study was the *B*.*thuringiensis* 4.0718(CCTCC No.M200016). It was grown on LB liquid broth (pH 7.6) consisting of polypeptone (1%), yeast extract (0.5%), NaCl (1%) and distilled water at 30°C with shaking at 200 rpm for overnight cultures as a pre-culture. 400 ul pre-culture medium was inoculated into 20 ml of LB medium and grown to OD = 1.0 at 30°C for 2.5–3 h.

### DNA extraction and testing

Total genome DNA from *B*.*thuringiensis* strain 4.0718 was isolated with Bacterial DNA Kit (OMEGO, USA), and look up instruction about detailed extraction process. DNA was quantitated using a Nanodrop 2000 (Thermo scientific) and homogeneity evaluated through agarose gel electrophoresis (1% agarose). The samples were sent for sequencing to Nextomics Biosciences, Wu Han. Sequencing was done by Illumina Hiseq 2000. The Nextomics provided the tested library concentration by qPCR was 246.5 nmol/L, and the analyzed fragment size by Agilent 2100 Bioanalyzer was 609 bp.

### Genome assembly and completion

Soap De novo alignment tool was used to clean data. The processes of assembly consisted of the following steps. (1) The scaffolds were compared with NCBI of nr/nt library to obtain the size and location of each scaffold, and defining which scaffold was chromosome sequence, which was plasmid sequence. (2) Selected the closest species (*Bacillus thuringiensis serovar kurstaki* str. HD73) as the reference genome (16s rDNA similarity 100%, data not shown) and download chromosomal genome. (3) Take the 199 scaffolds paste into different search interface using Gene Construction Kit 3.0, and since part of the sequence at both ends of each scaffold may be have matched with another scaffold, seek for which other scaffold is able to overlap. (4) Use the DNAman assembly software to take overlap section' scaffolds and assembled into longer scaffold. (5) Design PCR primer to repair the gap between scaffolds and complete the assembly of scaffolds.

### Analysis of genome structure and function

Annotation of genome contains structural and functional annotation. Prodigal was used to genes prediction have proved to be very fast, and have high-quality gene structural and translation initiation site prediction. The non-coding RNA also be analyzed by used the related software (WebMGA, tRNAscan-SE-1.3.1). Functional annotation of the identified genes was employing BLAST tools search against protein sequence database, which include KEGG, COG, SwissProt and NR. And then the genes of annotated molecular function, biological process, and cellular component could be received by means of online website Uniprot KB. In order to further analyses function of these genes, RAST and BASYs software could be applied in subsystem and cluster of orthologous group (COG) functional categories for proteins that were obtained from the completely sequenced genome.

### The analysis of Liquid Chromatography-Tandem Mass Spectrometry for whole-cell proteins

The process of sample preparation, extraction and digestion of whole-cell proteins, and 2D nano-LC-MS/MS (ThermoFisher, USA) analysis are using previously described methods [15].

## Results

### Whole genome sequencing, assembly, and functional analysis

We performed machine sequencing after library construction. Whole genome sequencing was conducted using shotgun strategy combined with high-throughput sequencing systems (Hiseq 2000), and such technique produced 23,548,110 high quality reads with an average length of 99. All the paired reads were assembled using SOAPdenovo (version 2.04), which resulted in 639 contigs (each contig has >100 bp). The results of the assembled high-quality data were as follows: bacterial genome size was 6.3 Mb; N50 was 93 kb in size; the maximum length was 222.9 kb; the GC content was 34.7%; and more than 2 kb contigs of 187 bp were obtained. After initial assembly, the resulting number of scaffolds is 199. *B*. *thuringiensis serovar kurstaki* strain HD73 was selected as a reference for analyzing the 199 scaffolds and for stitching these scaffolds by Gene Construction Kit 3.0 and DNAman software. A total of 15 Gaps were found in the process of assembly. Stitching was performed by primer walks and long-distance PCR amplification. The assembled genome size of the chromosomes is 5,641,982 bp. Seven plasmids were also detected in *B*. *thuringiensis* 4.0718, and a high degree of similarity was found among pHT7, pHT11, pHT8–1, pHT8–2, pHT73, pHT77, and pCT281 (Table 1). The pCT281 failed to assemble. The assembly results showed other information, including ncRNA (Table 2) and a repeat sequence.

**Table 1:** The partial information of plasmid-assembled.

Name	Sizes	GC	CDSs	High homologous plasmids
A	70,519	30.4%	95	pHT77
B	18,012	33.6%	11	pHT73
C	14,953	31.1%	23	pHT11
D	8,546	30.9%	10	pHT8–1
E	8,343	29.7%	1	pHT8–2
F	5,447	32.6%	7	pHT7

The data of these plasmids received by the RAST online website.

**Table 2:** The results of ncRNA prediction.

Type	Copy	Average length	Total length
tRNA	58	74 bp	4,300 bp
5S rRNA	12	114 bp	1,368 bp
16S rRNA	12	1,539 bp	18,468 bp
23S rRNA	12	2,919 bp	35,033 bp
Total	94		59,169 bp

The prediction of ncRNA by using WebMGA and tRNAscan-SE-1.3.1 software.

The 6,797 coding sequences (CDSs) have been searched by Prodigal.v2_60 software. The total CDS was 5,243,835 bp, which accounts for 83% of the genome. Analysis of the data showed that *B*. *thuringiensis* 4.0718 contained a total of 6,747 CDSs with encoded messages; 72.93% of the CDSs were associated with protein-coding functions, whereas 27.07% of CDSs have unknown function. RAST and BASYs software are applied in subsystem and cluster of orthologous group (COG) functional categories for proteins that were obtained from the completely sequenced genome. These CDSs were classified using the abovementioned software to determine the specific functions of each CDS in the metabolic process.

### Primary comparison analysis in genomic and proteomic data

**Fig 1:**
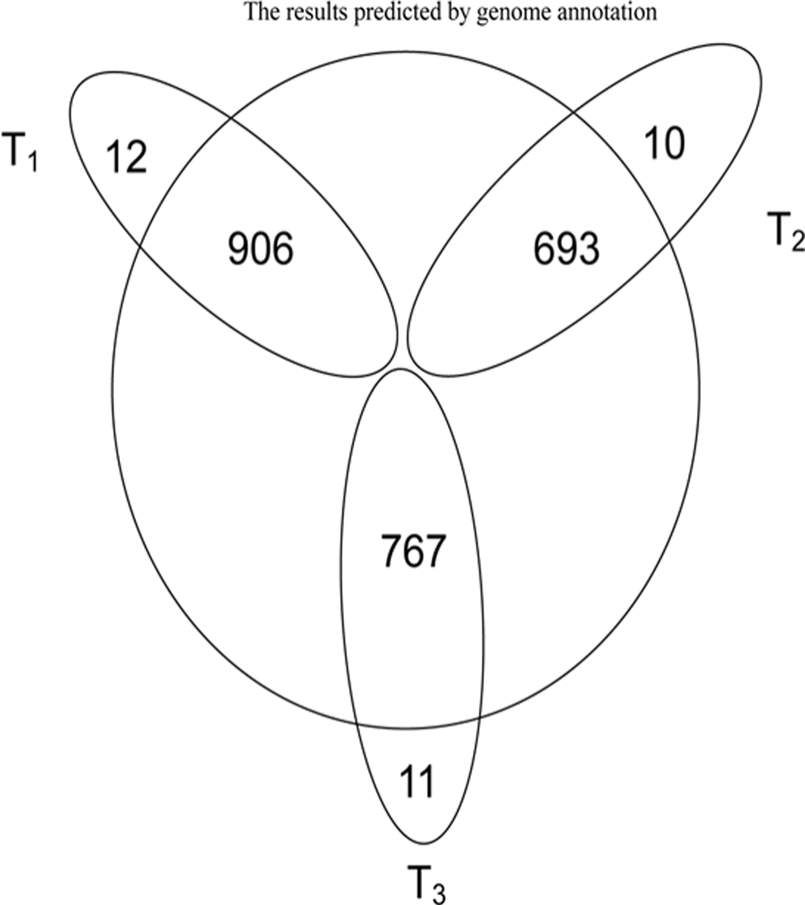
Protein identified of phases T1, T2 and T3 by mass spectrometry compared with the results of genome annotation. The part of circle outer represents the proteins that can be detected by LC-MS/MS but can not be searched from the results of the genome annotation.

**Fig 2:**
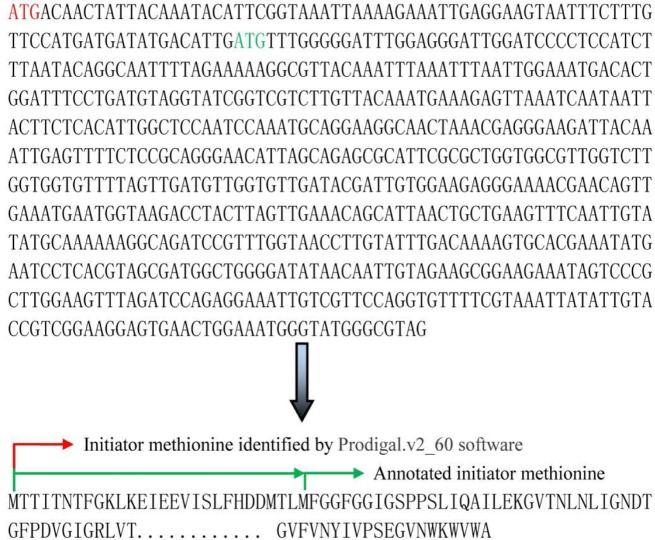
Analysis of start codon and initiator methionine for acetate CoA-transferase, alpha subunit. Red arrow, initiator methionine, as identified by Prodigal.v2_60 software. Green arrow, initiator methionine of the protein identified by LC-MS/MS through received uniprot blast. These initiator methionines were determined from *B*. *thuringiensis subsp*. *konkukian* (strain 97–27) and *B*. *thuringiensis serovar kurstaki* strain T03a001, respectively.

*B*. *thuringiensis* 4.0718 protein expression in proteomics was analyzed. Shaoya Huang has already performed this proteomics experiment in our laboratory, and thus, the analysis in this section mainly refers to the related proteomics analysis obtained from his study. The results from his study were compared with our genome annotation results. In Shaoya Huang’s research, 1,201 (1,034), 728 (662), and 854 (851) proteins at the T1 (10 h), T2 (20 h), and T3 (32 h) phases were screened, respectively, in two duplicate experiments. After deleting redundant proteins from different batches of *B*. *thuringiensis* subspecies, 918, 703, and 778 proteins in the T1, T2, and T3 phases were identified, respectively [15]. A total of 1,480 proteins were identified from the three phases, which accounted for 21.94% of the total protein post-genome annotation. Protein expression quality percentages at the three respective stages were 13.57%, 10.39%, and 11.50% of the encoded sequence of the *B*. *thuringiensis* 4.0718 genome. Upon comparison analysis, only three pesticidal Cry proteins (Cry2Aa, Cry1Aa, and Cry1Ac) were detected in his study. However, the genome annotation result shows that *B*. *thuringiensis* 4.0718 also contained other pesticidal Cry proteins, such as Cry2Ab and Cry1Ia (Table 3). By analyzing the positions of these five Cry protein genes in the genome, these genes were found to be located in two plasmids, and no Cry protein genes have been detected in the chromosome genome. Comparing the LC-MS/MS results to match the genome annotation results, few proteins were found each time we were unable to find or match the LC-MS/MS with the genome annotation results (Fig. 1, Tables 4 and 5). Generally, the unmatched proteins were mainly at different locations of the initiator methionine (Fig. 2). Therefore, the predicted result altered the number of methionine. Methionine also served an important function in the formation of either intra- or inter-chain disulfide bonds. Eventually, the analysis of the spatial structure of the protein will reveal differences.

**Table 3:** The comparative analysis of insecticidal activity substances in *B*.*thuringiensis* 4.0718 in genomics and proteomics.

Insecticidal activity substances description	Gene retrieval	LC-MS/MS detection
Pesticidal crystal protein(Cry2Aa, Cry2Ab, Cry1Aa,Cry1Ac, Cry1Ia)	+	+
Hemolytic enterotoxin	+	+
Non-hemolytic enterotoxin	+	+
Vegetative insecticidal protein Vip3V	+	+
Immune inhibitor A	+	+
Chitinase B	+	+
Hemolysin BL-binding component	+	-
Hemolysin BL lytic component L1	+	-
Hemolysin BL lytic component L2	+	-
Cytotoxin K	+	-
Bacteriocin	+	-
Alveolysin	+	-
Exoenzyme C3	+	-
SpoIISA like protein	+	-
help protein(ORF1, etc)	+	-

+, The insecticidal activity substance genes or proteins can be searched.

-, The insecticidal activity substance genes or proteins can not be searched.

**Table 4:** The results of the Mass spectrometry-based proteomic data compare with genome annotation data.

Type	T1	T2	T3
A	12	10	11
B	48	42	36

A, not found.

B, not match.

**Table 5:** The statistics of not found proteins for the result of genome annotation with comparison the result of the LC-MS/MS searched.

T1	T2	T3
Repetitive glutamine-rich protein	Staphylococcal nuclease homologue	Mobilization protein
Mername-AA019 peptidase	RapB	Spore germination protein BC
Baseplate hub protein	Mobilization protein	Sensor histidine kinase, sporulation kinase A
Tn7-like transposition protein C	Non-ribosomalpeptide synthase	Mername-AA019 peptidase
PXO1 ORF14-like protein	Mername-AA019 peptidase	Putative metalloprotein chaperonin subunit
Cytosine-specificmethyltransferase	Zwa6	Coenzyme PQQ synthesis protein
Spore germination protein BC	CapD	Beta-lactamase regulatory protein 1
Sulfurtransferase DndC	Putativeuncharacterized protein ORF179	TRNA(5-methylaminomethyl-2-thiouridylate)-methyltr-ansferase
SEC-C motif domain protein	Tn554-related, transposase C	SEC-C motif domain protein
Peptidase M48 Ste24p	Antilisterial bacteriocin (Subtilosin) production	Hippurate hydrolase
Putative uncharacterized protein yfjA		
ISSep1-like transposase		

### Comparative analysis of insecticidal activity substances

Given the interest on the pathogenesis of *B*. *thuringiensis* 4.0718, we determined proteins of related pathogenesis that are abundant in this species. Up to 39 CDSs were found in the genome, and such sequences were related to pathogenesis. These CDSs include pesticidal Cry protein, hemolytic enterotoxin, hemolysin BL, vegetative insecticidal protein Vip3V, cytotoxin K, bacteriocin, alveolysin, and exoenzyme C3. Pesticidal Cry proteins and ORF1 were encoded by plasmid. The plasmid and chromosome contained genes that can encode hemolytic enterotoxin. All the other toxins were encoded by chromosome. The LC–MS/MS results also showed different genome annotation outcomes in the substances with insecticidal activity (Table 3). Hemolysin BL, which exists in the chromosome and plasmid, was also detected by LC–MS/MS. This toxin has various characteristics that are similar to those of previously described enterotoxin preparations; hemolysin BL was proposed to be identical to the multi-component diarrheal toxin that is described by Thompson et al. [25] and Bitsaev and Ezepchuk [26]. Hemolysin BL comprises three components (B, L1, and L2), which are required for its activity. None of the individual components exhibited any toxic activity [27]. Hemolysin BL and lytic components L1 and L2 were not detected by LC–MS/MS. Therefore, the results of these were able to supports this conclusion about the *B*. *thuringiensis* insecticide can be safely used.

### Comparative analysis of spore formation and regulation

**Fig 3:**
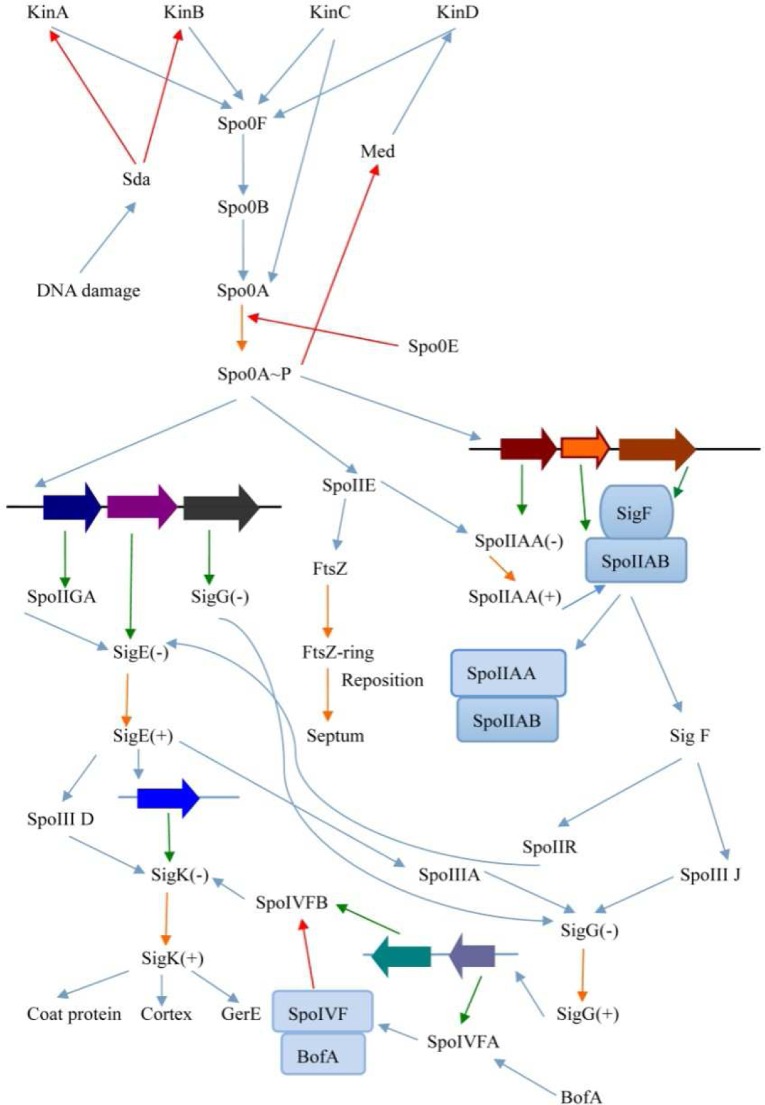
Complete circuit diagram of key regulatory genes in sporulation. Spo0A is a key protein that directs the transcriptional regulation of downstream gene, including asymmetric division and the expression of SigF and SigE, which are special transcription factors of pre-spore and mother cell, respectively. The two transcription factors decide the regulation of the development of spore. Red line, inhibition; Orange line, activation; Green, translation.

The response of *B*. *thuringiensis* 4.0718 under stressful conditions was determined. This species contained sufficient genetic messages for environmental adaptation. For example, under conditions in which the survival of the bacterium is impossible, this bacterium can initiate a developmental pathway that leads to the formation of dormant endospores. Gene function prediction results showed that the products of 172 CDS participate in the synthesis and regulation of endospores. The gene with related spore formation has been analyzed using related bioinformatics software, whereas a regulatory network was constructed for some spore-forming proteins (Fig. 3). Construction of this regulatory network mainly referred to the report on the key genes of spore formation and regulation, and such processes determine the presence or absence of these genes from the genome annotation results. Finally, the regulatory network diagram was completed. The figure mainly shows the regulatory network of several key proteins that were expressed during sporulation. Such proteins include sporulation initiation regulatory protein Spo0A and the pre-spore- and mother cell-specific proteins, namely, SigF and SigE, respectively. Analysis results of spore formation and regulatory proteins were also compared (Table 6). Table 6 shows that the partly sporulation-related proteins and those associated with regulation of spore formation weren't detected. Studies on the genes encoding such proteins will establish more perfect spore formation and regulation networks.

**Table 6:** The differential expression analysis of sporulation and regulation proteins related to the spore formation in genomic and proteomic.

Functional classification	The results of genome annotated	The results of LC-MS/MS searched
**Small acid-soluble spore proteins**	*SspI*, *SspE*, *SspB*, *SspK*, *SspN*	*SspI*, *SspE*, *SspB*, *SspK*, *SspN*, *SspP*
	*SspP*,SspO,*SspH1*, SspH2, Sasp-C3	*SspH1*, *Sasp-C5*, *Tlp*
	*Sasp-C5*, Sasp-1, Sasp-2, *Tlp*	
**Sporulation proteins**	*Spo0A*, *Spo0J*, *Spo0F*, *Spo0M*,Spo0B	*Spo0A*, *Spo0J*, *Spo0F*, *Spo0M*
	SpoIIP, SpoIIAA, SpoIIAB, SpoIIM	*SpoIIQ*, *SpoIIE*
	*SpoIIQ*, SpoIID, SpoIIR, *SpoIIE*	
	SpoIIIAA, SpoIIIAB, SpoIIIAC	*SpoIIIAG*, *SpoIIIAH*, *YidC(SpoIIIJ)*
	SpoIIIAD, SpoIIIAE, SpoIIIAF, *SpoIIIAG*	
	*SpoIIIAH*, SpoIIID, *YidC (SpoIIIJ)*	
	*SpoIVA*, SpoIVB, SpoIV, SpoIVFA	*SpoIVA*
	SpoIVFB	
	SpoVAA, SpoVAB, SpoVAC, SpoVAD	*SpoVD*, *SpoVR*, *SpoVS*, *SpoVT*
	SpoVAE, SpoVAF, SpoVB, *SpoVD*	
	SpoVE, SpoVFA, SpoVFB, SpoVG	
	SpoVK, *SpoVR*, *SpoVS*, *SpoVT*	
	SpoVAEA, SpoVAEB	
	*SpoVID*, SpoVIF	*SpoVID*
**Spore coat proteins**	*CotB*, CotC, CotZ, CotD, *CotE*, *CotH*	*CotB*, *CotE*, *CotH*, *CotX*, *CotM*, *GerQ*
	CotF, *CotX*, *CotM*, *GerQ*, SpsI, SpsK	*CotW*
	TasA, ExsA, *CotW*	
**Spore germination proteins**	GerA, GerB, GerC, *GerE*, *GerM*, GerPA	*GerE*, *GerM*, *GerPC*, *GerPE*
	GerPB, *GerPC*, GerPD, *GerPE*, GerPF	
	GerIA, GerBA, GerBB, YndE, YfkR	
	YaaH	

The italic represents contains both gene encoding the protein and can be detected by LC-MS/MS; The black font represents contains the gene encoding the protein but can not be detected by LC-MS/MS.

### Prediction of insecticidal Cry protein interaction network

**Fig 4:**
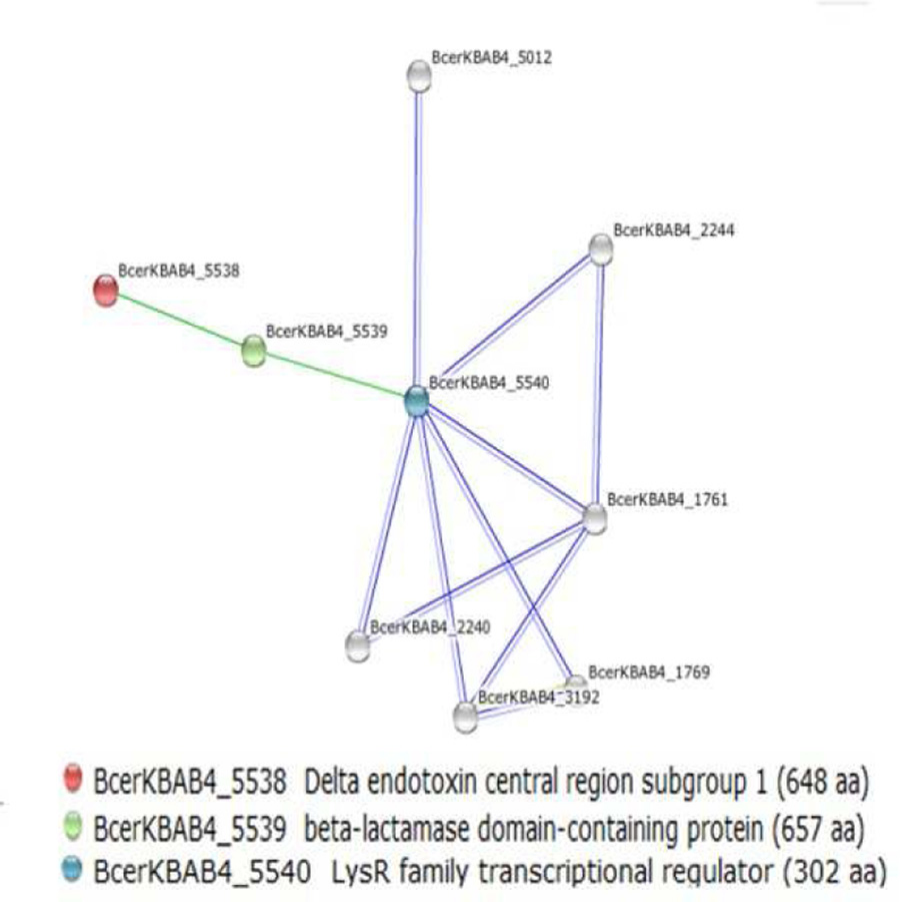
Analysis of insecticidal Cry protein regulation network. The figure exhibits that the insecticidal Cry protein interacts with beta-lactamase type II. Beta-lactamase type II is also regulated by LysR family transcriptional regulator.

The genome annotation results showed the presence of five different *cry* genes in *B*. *thuringiensis* 4.0718. These *cry* genes include *cry1Aa*, *cry1Ac*, *cry1Ia*, *cry2Aa*, and *cry2Ab*. The regulation and expression of these *cry* genes were analyzed through the related bioinformatics online website (String 9.1). These five *cry* genes share a common delta endotoxin central region subgroup 1, which can interact with beta-lactamase domain-containing protein (Fig. 4). The protein–protein interaction network scheme implies that beta-lactamase serves an important function in the activity of insecticidal Cry proteins. Beta-lactamase domain-containing protein belongs to beta-lactamase type II, which is a metalloprotease. Fig. 4 shows that this protease is regulated by a LysR family transcriptional regulator. However, the constructed regulation network was based on *B*. *weihenstephanensis*. Similar amino acid sequences of beta-lactamase domain-containing protein and LysR family transcriptional regulator were not found in *B*. *thuringiensis* 4.0718. However, beta-lactamase domain-containing protein and LysR family transcriptional regulator were detected by LC–MS/MS. Therefore, an experiment should be performed to confirm this regulatory network.

## Discussion

In this study, the whole genome of *B*. *thuringiensis* 4.0718 was successfully assembled based on the genome sequence drafted with next-generation sequencing technology. In the process of assembly, *B*. *thuringiensis serovar kurstaki* strain HD73 was selected as the reference genome for homologous analysis because this strain has high sequence similarity with *B*. *thuringiensis* 4.0718, as revealed by 16s rRNA analysis. However, *Bacillus thuringiensis* subsp. *kurstaki* strain HD73 has only contains one endotoxin gene, the *cry1Ac* gene, was found to be harbored in pHT73 [24]. However, *B*. *Thuringiensis* 4.0718 have had five *cry* genes and distribute into two different plasmids.

After the initial assembly, 15 Gaps were found in the chromosomal genome. Primer walks and long-distance PCR amplification were used to suture these gaps. In the chromosome assembly, the genome size and gene number are 5,641,982 bp and 5,985, respectively, and the GC content was 34.7%. Seven plasmids in *B*. *thuringiensis* 4.0718 showed a high degree of similarity with pHT7, pHT11, pHT8–1, pHT8–2, pHT73, pHT77, and pCT281.

To apply the obtained information on the genome, genome annotation should be performed. Annotation is a multilevel process used to define both structural and functional properties of a given sequence. In this study, Prodigal was selected as the software for gene prediction. Prodigal rapidly detected high-quality gene structures. Using this software, predicting translation initiation site was possible. The structure prediction also contained few false positives. WebMGA, tRNAscan-SE-1.3.1 and Tandem Repeat Finder were also selected to match the prediction of RNA and non-encoding sequence. In the process of function analysis, 27.03% of the total CDSs were located in an unknown functional region. Gene functional annotation is mainly used to predict genes of unknown function in homologous genes of known function. We failed to complete genome annotation because of imperfections in the database. Moreover, the analysis software has certain flaws that resulted in false negative occurrences during CDS analysis. Some genes in the *B*. *thuringiensis* genome are still unknown.

*B*. *thuringiensis* is an attractive species for biological insecticide research. Some encoded gene products in the *B*. *thuringiensis* 4.0718 strain have high insecticidal activity. These products include pesticidal Cry proteins (Cry2Aa, Cry2Ab, Cry1Aa, Cry1Ac, and Cry1Ia), hemolytic enterotoxin, non-hemolytic enterotoxin, hemolysin BL, vegetative insecticidal protein Vip3V, cytotoxin K, bacteriocin, alveolysin, exoenzyme C3, immune inhibitor A, chitinase B, and support proteins (e.g., ORF1). These encoded products directly or indirectly exhibit their insecticidal activity. Among these proteins, hemolysin BL is identical to the multi-component diarrheal toxin described by Thompson et al. [25] and Bitsaev and Ezepchuk [26]. The combination of all three components is also required for maximal activity exhibition. However, we failed to detect hemolysin BL and lytic components L1 and L2 by LC–MS/MS. Therefore, *B*. *thuringiensis* can be used as a safe insecticide. The regulation of sporulation was also analyzed. The gene function prediction result shows that the products of 172 CDS participated in the synthesis and regulation of endospores. We analyzed these CDSs, and a network of spore synthesis regulators was constructed. Fig. 3 shows the regulation mechanism underlying the functions of these key proteins during sporulation. This analysis revealed Spo0A～P, SigF, SigE(+), SigK(+) and SigG(+) plays a very important role in the process of spore formation regulatory network. Such mechanisms include those involved in Spo0A phosphorylation and regulation of a downstream gene to initiate sporulation, and the mechanisms underlying the expression of regulatory factors SigF and SigE in the pre-spore and mother cell, respectively. Subsequently, an interaction network of Cry proteins was constructed. Fig. 4 indicates that beta-lactamase domain-containing protein may affect the expression or activity of Cry proteins. However, this aspect has not yet been reported in *B*. *thuringiensis*. Therefore, further experiment should be defined to affirm this effect.

In the current study, the proteome of *B*. *thuringiensis* 4.0718 strain was also analyzed at three phases (middle vegetative, early sporulation, and late sporulation) and compared with the genome annotation data [15]. Protein expression quantities at the three different stages accounted for 13.57%, 10.39%, and 11.50% of the *B*. *thuringiensis* 4.0178 genome encoded sequence. A total of 1,480 proteins were identified at the three phases and accounted for 21.94% of the total protein post-genome annotation. The protein expression quantity at a particular period was much lower than that which the genome can express. Many silent genes or the special low expression level of genes were found. Huang et al. reported only three Cry proteins (Cry1Aa, Cry1Ac, and Cry2Aa), whereas the genome annotation results showed the presence of other pesticidal Cry proteins, such as Cry2Ab and Cry1Ia, in *B*. *thuringiensis* 4.0718. Based on comparison results, these two Cry proteins were unexpressed. We analyzed the location of these two *cry* genes in the genome and found that these genes were located in the plasmid and near the origin of replication. Such areas did not include a gene family that can be detected by LC–MS/MS. Therefore, such a result was not due to the silent gene cluster. The nucleotide sequences of these Cry proteins were also analyzed. The transposon sequence may affect the expression of *cry1Ia* and *cry2Ab*. The *cry2Ab* was not expressed because of an incomplete promoter. The analysis results have provided ideas for our next study. On one hand, the transposon sequences can be knocked out to eliminate their effects on *cry1Ia* and *cry2Ab*. On the other hand, promoter replacement method enables the creation of a functional promoter for *cry2Ab* to detect its expression.

In the final comparison analysis, due to the limitation of LC-MS/MS analysis, such as, unable to detect the special low abundance proteins, the data analysis remains to be perfected, a few proteins each time to not find or mismatching with the genome annotation results. This result may involve the following aspects. Using the Notes software, we found some defects that failed to correctly recognize the start and stop codes for the complex DNA. Some of the MS-identified proteins may be coded through the unknown gene. Besides, the incomplete of gene itself or exist the internal stop codons so that the relevant code product does not exist. These results will provide clues for investigating proteins that fail to match with the genome annotation message. Such clues include the validation or correction of initiator methionine through N-terminally acetylated peptides, analysis of the unknown gene, and *B*. *thuringiensis* genome transformation by means of the methods of synthetic biology.

In the case of protein translational start sites, start codons are hard to predict as the sequence context is generally not well defined [28]. Protein co-translational or post-translational N-terminal acetylation process involves cleavage of initiator methionine by aminopeptidases followed by acetylation of the following α-amino acid by N-acetyltransferases [29]. Such N-terminally acetylated peptides can be detected by LC–MS/MS, and to be used for the validation or correction of initiator methionine in annotated protein coding genes. Research on microbial reduced genome is a key focus of synthetic biology. In general, an engineering cell with reduced genome can optimize the metabolic pathway and enhance the controllability of physiological traits. Therefore, the *B*. *thuringiensis* genome can be reduced using the methods of synthetic biology be likely to enhance the yield of insecticidal Cry proteins. These studies are beneficial for the full understanding of the *B*. *thuringiensis* genome sequence information. The applications of *B*. *thuringiensis* in insect control may also be enhanced. In conclusion, proteomic research relies on the results of genomic sequencing, in turn, also modified and supplied the results. In the next few years, the increasing development of high resolution and high throughput protein analysis technology and the invention of multiple marker compounds, will make the analysis of qualitative and quantitative for proteomic more accurate and complete. The data analysis of proteomic and genomic in combination with more conductive to reveal the relevance of functional genes encoding and proteins expression. And also has very important significant for our further understanding the formation of toxic proteins and spore, as well, the mechanism of insecticidal.
